# Transcriptomic and epigenomic insights into ovarian cancer: a bioinformatics perspective – a narrative review

**DOI:** 10.1097/MS9.0000000000004798

**Published:** 2026-02-20

**Authors:** Emmanuel Ifeanyi Obeagu

**Affiliations:** a Division of Haematology, Department of Biomedical and Laboratory Science, Africa University, Muatre, Zimbabwe; b Department of Molecular Medicine and Haematology, School of Pathology, Faculty of Health Sciences, University of the Witwatersrand, Johannesburg, South Africa

**Keywords:** bioinformatics, epigenomics, molecular biomarkers, ovarian cancer, transcriptomics

## Abstract

Ovarian cancer is a highly heterogeneous malignancy with complex molecular underpinnings that extend beyond genomic mutations to encompass transcriptomic and epigenomic alterations. Advances in next-generation sequencing and bioinformatics have enabled comprehensive profiling of gene expression patterns, non-coding RNAs, DNA methylation, histone modifications, and chromatin accessibility, offering novel insights into ovarian tumor biology. Transcriptomic analyses reveal dysregulated coding and non-coding RNA networks, while epigenomic studies uncover epigenetic modifications that regulate gene expression and chromatin structure, together shaping the cancer phenotype. The integration of transcriptomic and epigenomic data through sophisticated bioinformatics pipelines allows the identification of key regulatory networks and molecular subtypes, enhancing our understanding of ovarian cancer heterogeneity and progression. Bioinformatics tools facilitate differential expression analysis, epigenetic mapping, and multi-omics data integration, revealing potential biomarkers and therapeutic targets. These approaches have also illuminated mechanisms of chemoresistance and immune evasion, providing avenues for personalized therapy and improved patient stratification. Datasets will be critical to harness the full potential of transcriptomic and epigenomic research. Ultimately, bioinformatics-driven insights into the ovarian cancer transcriptome and epigenome promise to inform early diagnosis, prognostication, and the development of targeted therapies, advancing precision oncology in this lethal

## Introduction

Ovarian cancer remains one of the most lethal gynecological malignancies worldwide, largely due to its asymptomatic nature in early stages and the resulting late diagnosis. Despite advances in surgical techniques and chemotherapy, the overall survival rate for ovarian cancer patients has remained disappointingly low. This poor prognosis is partly attributed to the complex molecular heterogeneity of ovarian tumors, which poses significant challenges for early detection, treatment, and prognosis. Traditional genomic analyses focusing on somatic mutations and copy number variations have provided important insights but fail to capture the full spectrum of molecular alterations driving ovarian cancer. As such, there is a growing recognition that understanding transcriptomic and epigenomic changes is essential for unveiling the intricate regulatory networks that underlie tumor initiation, progression, and therapeutic resistance^[^1,2^]^. Transcriptomics refers to the comprehensive study of RNA molecules expressed in a cell or tissue at a given time, including messenger RNA (mRNA), microRNA (miRNA), long non-coding RNA (lncRNA), and other non-coding RNA species. These transcripts regulate cellular function through diverse mechanisms, from coding for proteins to modulating gene expression post-transcriptionally. In ovarian cancer, transcriptomic analyses have revealed aberrant expression patterns that disrupt key cellular pathways such as cell cycle control, apoptosis, DNA repair, and angiogenesis. Moreover, non-coding RNAs have emerged as crucial regulators of gene expression and tumor biology, often acting as oncogenes or tumor suppressors. Investigating these transcriptomic landscapes provides dynamic insights into tumor behavior beyond static DNA mutations^[^3,4^]^.

**HIGHLIGHTS**

- Integrates transcriptomic and epigenomic data to unravel ovarian cancer mechanisms.
- Highlights bioinformatics tools for molecular profiling.
- Identifies biomarkers for prognosis.
- Explores therapeutic targets.
- Discusses clinical translation challenges.

Epigenomics encompasses heritable modifications that influence gene expression without altering the underlying DNA sequence. Key epigenetic mechanisms include DNA methylation, histone modifications, and chromatin remodeling, which collectively regulate chromatin accessibility and transcriptional activity. In ovarian cancer, aberrant DNA methylation patterns frequently silence tumor suppressor genes, while dysregulated histone modifications alter the expression of oncogenes and other critical regulators. These epigenetic changes contribute to tumor heterogeneity, metastasis, and resistance to chemotherapy. Importantly, unlike genetic mutations, epigenetic modifications are reversible, presenting unique therapeutic opportunities^[^5^]^. The emergence of high-throughput sequencing technologies, such as RNA sequencing (RNA-seq), whole-genome bisulfite sequencing, and chromatin immunoprecipitation sequencing (ChIP-seq), has transformed transcriptomic and epigenomic research. These platforms generate vast amounts of data, enabling comprehensive molecular profiling of ovarian tumors at unprecedented resolution. However, the complexity and volume of data necessitate advanced bioinformatics tools for processing, analysis, and interpretation. Bioinformatics facilitates identification of differentially expressed genes and RNAs, detection of epigenetic marks, functional enrichment analysis, and integrative multi-omics approaches, all essential for translating molecular findings into biological understanding and clinical application^[^6,7^]^.

The integration of transcriptomic and epigenomic data offers a systems biology perspective, uncovering how epigenetic modifications shape gene expression patterns and influence tumor phenotypes. Such integrative analyses can identify molecular subtypes of ovarian cancer with distinct prognoses and therapeutic vulnerabilities. Additionally, they help to elucidate mechanisms of drug resistance and immune escape, guiding the development of targeted therapies and combination treatment strategies. Bioinformatics approaches including network modeling, machine learning, and multi-omics factor analysis play a crucial role in these endeavors, highlighting the intersection between computational biology and oncology^[^8,9^]^.

Cancer remains a leading cause of morbidity and mortality worldwide, driven by the complex interplay of genetic, epigenetic, and environmental factors that disrupt normal cellular regulatory mechanisms and promote unchecked proliferation, invasion, and metastatic dissemination. Global estimates continue to show rising cancer incidence, with breast, lung, colorectal, prostate, and hepatocellular carcinomas among the most prevalent and clinically burdensome malignancies across diverse populations. Advances in molecular oncology have revealed that tumor development is governed not only by genomic alterations but also by widespread transcriptomic reprogramming and epigenetic dysregulation that reshape cellular identity and microenvironmental interactions. Within this broader landscape, ovarian cancer stands out as one of the deadliest gynecologic cancers, largely due to its molecular heterogeneity, clinically silent progression, and frequent late-stage presentation. Understanding ovarian cancer within the context of global cancer biology underscores the importance of high-resolution molecular profiling approaches to elucidate the regulatory networks that underlie its aggressive behavior and therapeutic resistance^[^6,10,11^]^.

## Aim

The aim of this narrative review is to comprehensively explore the transcriptomic and epigenomic landscapes of ovarian cancer through the lens of bioinformatics.

## Methods

This narrative review was developed through a structured and transparent approach designed to ensure comprehensive coverage of transcriptomic, epigenomic, and integrative bioinformatics studies in ovarian cancer. Although not conducted as a systematic review, the methodology followed a clear framework to enhance rigor and reproducibility. The literature search was performed across several major scientific databases, including PubMed, Scopus, Web of Science, and Google Scholar, covering the period from January 2010 to January 2025. This timeframe was selected to capture both foundational molecular studies and the most recent advances in high-throughput transcriptomic and epigenomic profiling. Search terms were combined using Boolean operators and relevant MeSH terminology and included expressions related to ovarian cancer, transcriptomics, RNA sequencing, epigenomics, DNA methylation, chromatin remodeling, single-cell technologies, multi-omics integration, prognostic signatures, and therapeutic resistance.

During the initial screening process, titles and abstracts were reviewed to identify studies relevant to the molecular, bioinformatic, and clinical themes of the review. Full texts of potentially eligible articles were then examined to evaluate methodological robustness and relevance to the overarching objective of synthesizing current knowledge on gene expression and epigenetic regulation in ovarian cancer. Preference was given to peer-reviewed original research articles, meta-analyses, and authoritative reviews that employed validated omics platforms such as bulk RNA sequencing, single-cell RNA sequencing, ATAC-seq, DNA methylation arrays, chromatin accessibility assays, and integrated multi-omics frameworks. Studies that lacked methodological clarity, did not involve ovarian cancer, or focused solely on unrelated model systems were excluded, unless they provided essential mechanistic insights applicable to ovarian cancer biology.

Data from the included literature were extracted and synthesized thematically rather than quantitatively. Particular attention was paid to the methodological approaches used in transcriptomic and epigenomic analyses, including differential expression pipelines, integrative bioinformatic tools, network-based modeling, machine learning applications, and pathway enrichment strategies. Findings were grouped into conceptual themes that reflected the major domains of contemporary ovarian cancer research, such as transcriptomic dysregulation, epigenetic alterations, non-coding RNA networks, multi-omics integration, single-cell and spatial insights, and mechanisms underlying chemoresistance and therapeutic response. Divergences across studies were critically evaluated by considering differences in sample characteristics, population variability, analytical pipelines, and validation strategies.

Although a formal risk-of-bias assessment was not applied due to the narrative nature of this review, methodological transparency, statistical rigor, reproducibility indicators, and validation frameworks were considered when weighing the strength of evidence. Studies utilizing publicly available datasets such as TCGA, ICGC, and GEO were prioritized, particularly when accompanied by independent cohort validation. Throughout the process, careful attention was given to ensuring balanced interpretation of findings, acknowledging study limitations, and maintaining consistency in referencing key literature. As this article synthesizes previously published data, no ethical approval was required. The final narrative represents an integrated and critically appraised overview of the current transcriptomic and epigenomic landscape of ovarian cancer, guided by a transparent and methodologically sound literature review process.

### Transcriptomic insights into ovarian cancer

Transcriptomic profiling has emerged as a powerful approach to unravel the dynamic gene expression changes underlying ovarian cancer development and progression. By analyzing the complete set of RNA transcripts – both coding and non-coding – researchers have gained critical insights into the molecular pathways that drive tumor initiation, growth, and metastasis. Early transcriptomic studies, primarily using microarrays, identified key differentially expressed genes involved in cell cycle regulation, apoptosis, DNA repair, and angiogenesis, laying the groundwork for understanding ovarian tumor biology at the molecular level. More recently, RNA-seq has revolutionized transcriptomics by providing higher resolution, greater sensitivity, and the ability to detect novel transcripts and alternative splicing events, significantly expanding our knowledge of ovarian cancer transcriptomes^[^6,10^]^.

One notable finding from transcriptomic analyses is the identification of distinct molecular subtypes of ovarian cancer, which differ in their gene expression patterns, clinical outcomes, and responses to therapy. For example, high-grade serous ovarian carcinoma (HGSOC), the most common and aggressive subtype, has been classified into multiple transcriptional subtypes with unique biological characteristics such as immunoreactive, proliferative, mesenchymal, and differentiated profiles. These subtypes not only provide prognostic value but also suggest tailored therapeutic strategies by pinpointing pathway dysregulations specific to each group. Beyond protein-coding genes, non-coding RNAs – especially miRNAs and lncRNAs – have garnered considerable attention for their regulatory roles. Dysregulated miRNAs can act as oncogenes or tumor suppressors by targeting messenger RNAs for degradation or translational repression, while lncRNAs modulate gene expression through diverse mechanisms including chromatin remodeling and transcriptional interference^[^11,12^]^.

Moreover, transcriptomic studies have shed light on mechanisms of chemoresistance, a major challenge in ovarian cancer treatment. Differential expression of genes involved in drug metabolism, DNA damage repair pathways such as homologous recombination, and epithelial-to-mesenchymal transition processes have been associated with resistance to platinum-based chemotherapy. Additionally, transcriptomics has revealed the importance of the tumor microenvironment, highlighting the interactions between cancer cells and stromal or immune cells that influence tumor progression and therapeutic response. Single-cell RNA sequencing (scRNA-seq) is an emerging tool that further refines these insights by dissecting cellular heterogeneity within tumors and identifying rare cell populations that contribute to disease aggressiveness and resistance. Collectively, transcriptomic analyses provide a rich, nuanced view of ovarian cancer biology and offer promising avenues for biomarker discovery and development of novel therapeutic interventions (Table 1)^[^13,14^]^.

**Table 1:** Transcriptomic insights in ovarian cancer.

Category	Key findings	Representative genes/pathways	Clinical or biological implications
Differential gene expression	Dysregulation of cell cycle, DNA repair, EMT, angiogenesis pathways	CCNE1, MYC, RAD51, VEGFA	Supports tumor proliferation, genomic instability, invasion, and metastatic potential
Transcriptomic subtypes (TCGA)	Immunoreactive, proliferative, mesenchymal, and differentiated subtypes identified	Immune markers, ECM remodeling genes, proliferation-associated genes	Enables molecular classification linked to prognosis and treatment response
EMT and metastatic programs	Upregulation of EMT transcription factors and migratory-associated signatures	ZEB1, SNAI2, TWIST1, COL11A1	Drives peritoneal dissemination and chemoresistance
Immune-related signatures	Variable immune infiltration and cytokine signaling patterns	STAT3, IL6, CXCL9/10	Predictive of immunotherapy responsiveness and tumor immune evasion
Non-coding RNA regulation	Altered expression of microRNAs, lncRNAs, and circRNAs	miR-200 family, miR-21, MALAT1, H19, circHIPK3	Regulates EMT, apoptosis, drug resistance, and oncogenic signaling
Single-cell transcriptomics	Identification of intratumoral heterogeneity and chemoresistant subpopulations	CLIC3-high epithelial clusters; cancer stem-like cells	Reveals cell-state transitions and mechanisms of platinum and PARP inhibitor resistance
Metabolic reprogramming	Altered expression of metabolic enzymes and transporters	PDK1, LDHA, SLC family genes	Supports tumor survival under hypoxia and contributes to therapy resistance
Transcriptomic biomarker discovery	Identification of expression-based prognostic and predictive markers	HRD-related transcripts, immune activation signatures	Guides risk stratification and precision oncology approaches

### Epigenomic insights into ovarian cancer

Epigenomic alterations play a pivotal role in the initiation and progression of ovarian cancer by modulating gene expression without changes to the underlying DNA sequence. These heritable but reversible modifications include DNA methylation, histone modifications, and chromatin remodeling, all of which influence chromatin structure and accessibility. Aberrant DNA methylation patterns, particularly hypermethylation of tumor suppressor gene promoters, have been extensively documented in ovarian cancer. This epigenetic silencing contributes to loss of gene function involved in critical pathways such as cell cycle regulation, apoptosis, and DNA repair. For instance, hypermethylation of the BRCA1 promoter has been identified in a subset of ovarian tumors, mimicking the effects of BRCA1 mutations and conferring similar defects in homologous recombination repair^[^15,16^]^. Histone modifications, including methylation, acetylation, phosphorylation, and ubiquitination, constitute another layer of epigenetic regulation that influences gene transcription. Altered patterns of histone marks, such as reduced histone H3 lysine 27 trimethylation (H3K27me3) or increased histone acetylation, have been linked to dysregulated expression of oncogenes and tumor suppressor genes in ovarian cancer. Enzymes responsible for writing, erasing, or reading these histone modifications – such as histone methyltransferases, demethylases, and acetyltransferases – are frequently dysregulated, contributing to tumor progression and therapeutic resistance. These epigenetic enzymes have therefore emerged as promising targets for epigenetic therapies^[^17,18^]^.

Chromatin remodeling complexes, which alter nucleosome positioning and chromatin accessibility, further impact gene regulation in ovarian cancer. Mutations or altered expression of components of these complexes can disrupt normal chromatin architecture, facilitating aberrant transcriptional programs that promote oncogenesis. High-throughput epigenomic profiling techniques such as whole-genome bisulfite sequencing, ATAC-seq, and ChIP-seq have enabled comprehensive mapping of these epigenetic landscapes, revealing extensive heterogeneity among ovarian tumors. Integrating epigenomic data with transcriptomic profiles has improved the understanding of how epigenetic modifications orchestrate gene expression changes, ultimately driving ovarian cancer development, progression, and drug resistance (Table 2)^[^19,20^]^.

**Table 2:** Epigenomic insights in ovarian cancer.

Category	Key findings	Representative genes/mechanisms	Clinical or biological implications
DNA methylation alterations	Promoter hypermethylation of tumor suppressor genes; global hypomethylation	BRCA1, RASSF1A, MLH1; LINE-1 hypomethylation	Drives genomic instability, silences tumor suppressors, contributes to chemoresistance and tumor progression
Histone modifications	Dysregulation of activating and repressive histone marks	H3K27me3, H3K4me3, H3K9ac	Alters chromatin accessibility, impacts transcriptional regulation, and promotes oncogenic programs
Chromatin remodeling defects	Mutations and loss of function in chromatin remodelers	ARID1A, SMARCA4 (SWI/SNF complex)	Enhances enhancer reprogramming, supports aggressive phenotypes, and contributes to therapy resistance
Non-coding RNA-mediated epigenetic regulation	lncRNAs and miRNAs regulate chromatin and transcription	MALAT1, H19, miR-200, miR-214	Modulates gene expression, EMT, apoptosis, and chemoresistance through epigenetic mechanisms
Integrated epigenomic–transcriptomic signatures	Combined methylation and expression analyses reveal prognostic and therapy-related pathways	Methylation-regulated genes controlling DNA repair, apoptosis, and immune pathways	Facilitates patient stratification and predictive biomarker discovery
Chromatin accessibility and super-enhancers	Altered enhancer landscapes driving oncogene expression	PAX8, MYC, SOX8	Reveals lineage-specific dependency pathways and potential therapeutic targets
Epigenetic drivers of drug resistance	Epigenetic reprogramming contributes to chemoresistance and PARP inhibitor resistance	DNA repair genes, apoptosis regulators	Provides rationale for epigenetic-targeted therapies in combination with chemotherapy or PARPi

### Bioinformatics approaches for integrative analysis

The complex interplay between transcriptomic and epigenomic alterations in ovarian cancer necessitates sophisticated bioinformatics approaches capable of integrating diverse molecular datasets to generate holistic insights. Integrative analysis involves combining multiple layers of omics data – such as gene expression profiles, DNA methylation patterns, histone modifications, and chromatin accessibility – to uncover regulatory networks, identify molecular subtypes, and elucidate mechanisms driving tumor heterogeneity and progression. This systems biology perspective is crucial for understanding how epigenetic modifications influence transcriptional programs and contribute to oncogenesis^[^21,22^]^. A variety of computational frameworks and algorithms have been developed to facilitate multi-omics integration. Methods such as matrix factorization, canonical correlation analysis, and multi-omics factor analysis enable joint dimensionality reduction and correlation of heterogeneous datasets. Network-based approaches construct gene regulatory or protein–protein interaction networks by integrating expression and epigenetic data, revealing key driver genes or epigenetic regulators that may serve as potential therapeutic targets. Machine learning and clustering techniques are also extensively applied to classify ovarian tumors into molecular subtypes based on combined transcriptomic and epigenomic signatures, improving patient stratification and prognostic predictions^[^23,24^]^.

Several bioinformatics tools and platforms have been tailored for integrative multi-omics analysis in cancer research. Examples include iClusterPlus, which performs joint clustering of multi-omics data to discover tumor subgroups; MethylMix, which integrates DNA methylation and gene expression data to identify functional epigenetic alterations; and ELMER, which links distal enhancer methylation changes to transcriptional regulation. Visualization tools such as Circos plots and integrative genome viewers facilitate interpretation of complex data relationships. Despite advances, challenges remain in standardizing data preprocessing, handling batch effects, and addressing missing data across datasets. Ongoing development of robust, scalable, and user-friendly bioinformatics pipelines will be essential for translating integrative multi-omics findings into clinical applications for ovarian cancer^[^25,26^]^.

### Clinical implications

The integration of transcriptomic and epigenomic data in ovarian cancer has profound clinical implications, offering new avenues for early diagnosis, prognosis, and personalized treatment strategies. Molecular profiling of tumors using high-throughput sequencing technologies enables the identification of specific gene expression signatures and epigenetic alterations that distinguish ovarian cancer subtypes with differing clinical behaviors. These molecular subtypes can inform prognosis by predicting tumor aggressiveness, likelihood of metastasis, and patient survival outcomes more accurately than traditional histopathological classification alone^[^27,28^]^. Transcriptomic biomarkers, including coding and non-coding RNAs, have shown promise as minimally invasive diagnostic and prognostic tools. Circulating tumor RNAs and microRNAs detected in blood or other bodily fluids could serve as liquid biopsy markers, facilitating early detection and monitoring of disease progression or therapeutic response. Epigenetic alterations such as DNA methylation patterns are also being explored as biomarkers, given their stability and detectability in circulating tumor DNA. For instance, hypermethylation of specific gene promoters could serve as an early warning sign for ovarian cancer or indicate resistance to standard chemotherapies^[^29,30^]^.

Importantly, epigenetic modifications are reversible, making them attractive targets for therapeutic intervention. Epigenetic drugs, such as DNA methyltransferase inhibitors and histone deacetylase inhibitors, are currently being evaluated in clinical trials for ovarian cancer, either as monotherapies or in combination with chemotherapy, immunotherapy, or targeted agents. Furthermore, understanding the transcriptomic changes associated with drug resistance mechanisms helps guide the development of novel therapies aimed at overcoming treatment failure. Integrative multi-omics approaches also hold promise for personalized medicine by enabling tailored therapeutic regimens based on a patient’s unique molecular profile, ultimately improving clinical outcomes and reducing adverse effects^[^2,31^]^.

### Ovarian cancer diagnosis

The diagnosis of ovarian cancer relies on a combination of clinical assessment, serum biomarkers, imaging modalities, and confirmatory histopathology. Because early-stage disease is often asymptomatic and lacks specific clinical features, imaging plays a central role in detecting adnexal masses and determining their likelihood of malignancy^[^21^]^. Transvaginal ultrasound is the primary initial imaging tool due to its high sensitivity, real-time assessment of ovarian structure, ability to evaluate internal septations, papillary projections, and vascular flow patterns, and its utility in risk stratification through scoring systems such as the International Ovarian Tumor Analysis models. Computed tomography (CT) is widely used for staging purposes, providing detailed visualization of peritoneal dissemination, omental involvement, nodal enlargement, and distant metastases, which are critical for planning surgical cytoreduction^[^32^]^. Magnetic resonance imaging, with its superior soft-tissue contrast and functional sequences – including diffusion-weighted imaging – enhances lesion characterization and can help differentiate benign from malignant ovarian masses, especially in cases where ultrasound findings are indeterminate^[^28^]^. Positron emission tomography (PET), typically integrated with CT (PET/CT), offers metabolic imaging that improves detection of hypermetabolic malignant lesions, recurrence assessment, and evaluation of treatment response, although its role is often adjunctive rather than first-line in adnexal mass evaluation. Collectively, these imaging modalities form an essential component of the ovarian cancer diagnostic workflow, guiding clinical decision-making and ensuring timely identification of disease before proceeding to surgical exploration and comprehensive molecular characterization^[^33,34^]^.

### Future directions

The continued evolution of high-throughput sequencing technologies and bioinformatics methodologies promises to deepen our understanding of ovarian cancer’s molecular complexity and improve patient management. Future research should focus on expanding integrative multi-omics analyses that combine transcriptomic, epigenomic, proteomic, and metabolomic data to construct comprehensive molecular maps of ovarian tumors. Such holistic profiling will enhance the resolution of tumor heterogeneity, uncover novel regulatory mechanisms, and identify previously unrecognized therapeutic targets. Additionally, integrating spatial transcriptomics and epigenomics will provide crucial insights into tumor microenvironment dynamics and cellular interactions, further informing treatment strategies^[^6,35^]^. Advancements in single-cell sequencing technologies offer exciting opportunities to dissect intratumoral heterogeneity at unprecedented granularity. Applying single-cell transcriptomic and epigenomic analyses will enable the identification of rare cell populations, such as cancer stem cells or immune evasive clones, which play critical roles in therapy resistance and metastasis. These insights can guide the development of more effective, targeted therapies and inform strategies to prevent relapse. Moreover, longitudinal multi-omics profiling of patient samples during treatment will facilitate real-time monitoring of tumor evolution, enabling adaptive therapeutic interventions tailored to dynamic molecular changes^[^36,37^]^.

To translate these molecular insights into clinical practice, future efforts must also focus on developing standardized, robust bioinformatics pipelines, and user-friendly platforms that support data integration and interpretation across research and clinical settings. Establishing large, well-annotated ovarian cancer multi-omics datasets with linked clinical outcomes will be essential for validating biomarkers and predictive models. Furthermore, fostering interdisciplinary collaborations between computational biologists, clinicians, and pharmaceutical developers will accelerate the design of precision medicine trials and epigenetic therapies. Ultimately, these future directions aim to improve early detection, personalize treatment and enhance survival outcomes for ovarian cancer patients worldwide^[^38–40^]^.

## Conclusion

Transcriptomic and epigenomic analyses have profoundly expanded our understanding of ovarian cancer’s molecular underpinnings, revealing complex regulatory networks that drive tumor initiation, progression, and therapeutic resistance. Bioinformatics tools have been instrumental in deciphering these vast datasets, enabling the identification of molecular subtypes, key driver genes, and epigenetic alterations with potential diagnostic, prognostic, and therapeutic relevance. The integration of transcriptomic and epigenomic data offers a more comprehensive view of ovarian cancer biology than either approach alone, highlighting the interplay between gene expression and epigenetic regulation in shaping tumor behavior. These molecular insights have significant clinical implications, paving the way for personalized medicine approaches that tailor treatment based on individual tumor profiles. Biomarkers derived from transcriptomic and epigenomic signatures hold promise for early detection and monitoring, while epigenetic therapies offer novel avenues for overcoming drug resistance. Despite considerable progress, challenges remain in standardizing multi-omics analyses, validating biomarkers in clinical cohorts, and translating findings into effective treatments.

## Data Availability

There were no data generated as this is a narrative review article.

## Research registration unique identifying number (UIN)

No human subjects were involved in this study; hence, no registration was required.
